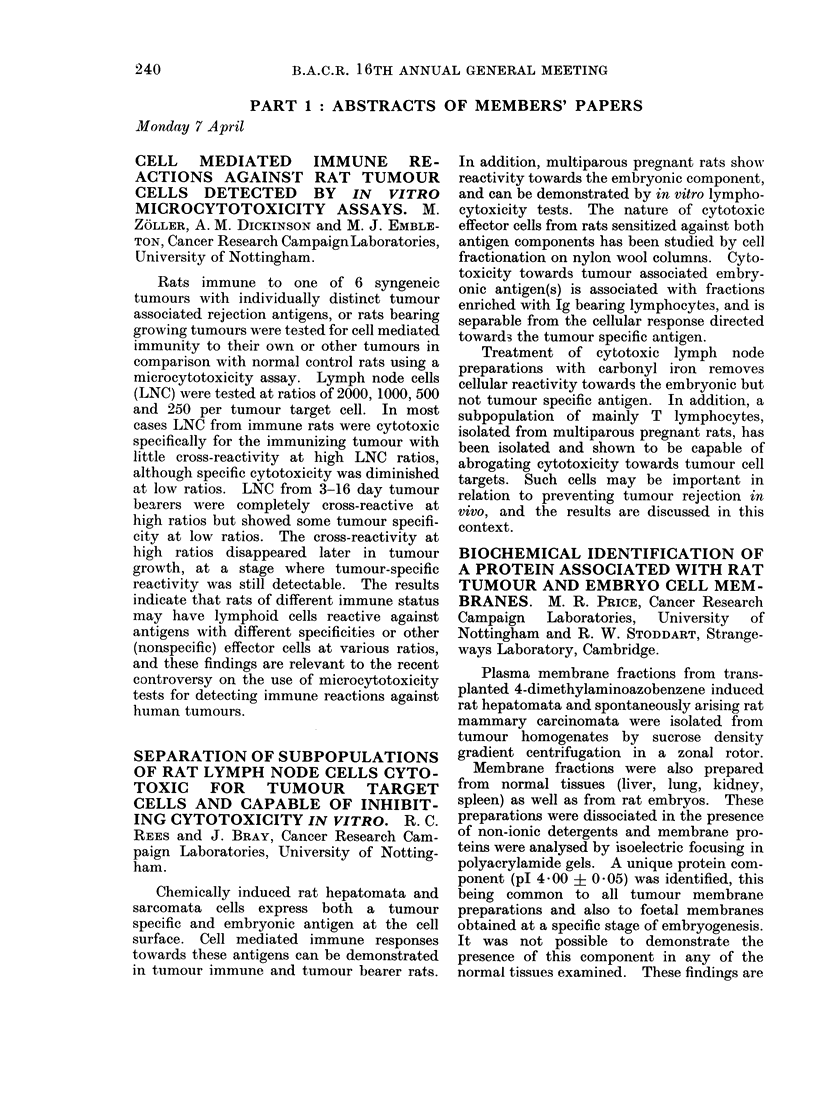# Proceedings: Separation of subpopulations of rat lymph node cells cytotoxic for tumour target cells and capable of inhibiting cytotoxicity in vitro.

**DOI:** 10.1038/bjc.1975.155

**Published:** 1975-08

**Authors:** R. C. Rees, J. Bray


### SEPARATION OF SUBPOPULATIONS OF RAT LYMPH NODE CELLS CYTOTOXIC FOR TUMOUR TARGET CELLS AND CAPABLE OF INHIBITING CYTOTOXICITY *IN VITRO*.

R. C. Rees and J. Bray, Cancer Research Campaign Laboratories, University of Nottingham.

Chemically induced rat hepatomata and sarcomata cells express both a tumour specific and embryonic antigen at the cell surface. Cell mediated immune responses towards these antigens can be demonstrated in tumour immune and tumour bearer rats. In addition, multiparous pregnant rats show reactivity towards the embryonic component, and can be demonstrated by *in vitro* lymphocytoxicity tests. The nature of cytotoxic effector cells from rats sensitized against both antigen components has been studied by cell fractionation on nylon wool columns. Cytotoxicity towards tumour associated embryonic antigen(s) is associated with fractions enriched with Ig bearing lymphocytes, and is separable from the cellular response directed towards the tumour specific antigen.

Treatment of cytotoxic lymph node preparations with carbonyl iron removes cellular reactivity towards the embryonic but not tumour specific antigen. In addition, a subpopulation of mainly T lymphocytes, isolated from multiparous pregnant rats, has been isolated and shown to be capable of abrogating cytotoxicity towards tumour cell targets. Such cells may be important in relation to preventing tumour rejection *in vivo*, and the results are discussed in this context.